# Spatial and Temporal Attention Modulate the Early Stages of Face Processing: Behavioural Evidence from a Reaching Paradigm

**DOI:** 10.1371/journal.pone.0057365

**Published:** 2013-02-28

**Authors:** Genevieve L. Quek, Matthew Finkbeiner

**Affiliations:** Department of Cognitive Science, ARC Centre of Excellence in Cognition and its Disorders, Macquarie University, Sydney, New South Wales, Australia; CEA.DSV.I2BM.NeuroSpin, France

## Abstract

A presently unresolved question within the face perception literature is whether attending to the location of a face modulates face processing (i.e. spatial attention). Opinions on this matter diverge along methodological lines – where neuroimaging studies have observed that the allocation of spatial attention serves to enhance the neural response to a face, findings from behavioural paradigms suggest face processing is carried out independently of spatial attention. In the present study, we reconcile this divide by using a continuous behavioural response measure that indexes face processing at a temporal resolution not available in discrete behavioural measures (e.g. button press). Using reaching trajectories as our response measure, we observed that although participants were able to process faces both when attended and unattended (as others have found), face processing was not impervious to attentional modulation. Attending to the face conferred clear benefits on sex-classification processes at less than 350ms of stimulus processing time. These findings constitute the first reliable demonstration of the modulatory effects of both spatial and temporal attention on face processing within a behavioural paradigm.

## Introduction

Of the many objects we encounter in the visual world, faces are perhaps the most biologically and socially significant. Accordingly, faces hold a particularly important status within the human visual system, eliciting specific neural responses in the Fusiform Face Area (FFA) [1]–[4], and readily capturing visual attention over other objects in a scene [5]–[8]. Although the relationship between attention and faces has been widely examined in the literature, much of the existing research has documented the effects of object- or task-based attention, in which subjects direct their attention to face- or non-face-stimuli according to the task instructions [9]–[12]. A comparatively unresolved question concerns how directing attention to the *location* of a face modulates the processing of this stimulus. Intriguingly, it is now well-established that face processing can in fact proceed in the near-absence of spatial attention. For example, Reddy and colleagues have shown that subjects’ ability to classify the sex or identity of peripheral faces does not suffer significantly when spatial attention is held centrally by a demanding discrimination task. Face performance in this dual task condition was not significantly different to when they explicitly attended to the peripheral face [13]–[15]. Similarly, we have reported elsewhere that the sex of a briefly presented masked face (called the prime) affects subjects’ classification of a subsequent target face to the same degree regardless of whether spatial attention had been captured to the prime’s location or elsewhere [16]. Comparative effects have been observed for face-fame judgement tasks [17]. However, where these behavioural studies might suggest face processing is carried out independently of attention, functional magnetic resonance imaging (fMRI) research has demonstrated that the allocation of attention to the region of space in which a face appears enhances the associated haemodynamic response in the FFA [18]–[21] (but see [13]). For example, Vuilleumier and colleagues [20] used a four placeholder crossed display to have subjects report identical matches for face or house stimuli. Prior to each block, participants saw a cue that indicated which pair of placeholders they should attend to (vertical or horizontal), and were instructed to ignore stimuli appearing in the uncued, irrelevant locations. The authors were thus able to compare the blood-oxygen-level-dependent (BOLD) response for spatially attended and unattended faces in the same physical location, finding greater activation in the FFA for the former. Similar effects have been reported elsewhere [18], [19], [21]. At present, this discrepancy in the face perception literature remains unresolved – is face processing carried out independently of spatial attention, as existing behavioural data might suggest [13]–[17]? Or are faces just more robust to manipulations of spatial attention than non-face stimuli [22]–[25]? We reconcile this issue in the present study by demonstrating for the first time reliable effects of both spatial and temporal attention on sex-classification processes within a behavioural task [16], [17], [26].

One possible explanation for the lack of observable attentional effects in previous behavioural studies relates to the measure they typically employ: button-press responses. Although widely used in cognitive research, we suggest that this discrete measure of cognitive processes may not be sufficiently sensitive to reveal the modulatory effects of spatial/temporal attention in face classification tasks. Sex-categorisation, for example, is extremely efficient. Even in the absence of overt gender cues (e.g. hairstyle or facial hair), subjects are able to discriminate the sex of a target very reliably and quickly [27], [28]. Accordingly, we might expect that any attentional benefit relating to this process would be difficult to detect, since performance is already very close to ceiling. Sreenivasan and colleagues have documented this, showing no attentional modulation of the face-specific event-related potential (ERP) known as the N170 for highly discriminable faces, but a clear attentional benefit on the same component when the perceptual quality of faces is degraded, effectively reducing the signal-to-noise ratio [29]. Given that attentional effects are clearest with degraded faces [29], it is reasonable to think that the modulatory effects of attention would be fleeting, if they are present at all, with non-degraded faces. With this in mind, we reasoned that the possibility of observing attentional effects would be the greatest during the earliest stages of stimulus processing, when perceptual evidence is still being accumulated. In support of this supposition are neurophysiological findings that spatial attention modulates the early stages of sensory processing for faces. For example, Jacques and Rossion [30] showed that spatial attention modulates visual processes effects as early as 80 ms after stimulus onset, as well enhancing the N170 component. Similarly, a recent study by Wijers and Banis [31] observed that directing subjects’ spatial attention to the location of a face enhanced the mean amplitude of early visual components P1 and N1 elicited by this stimulus. Taken together, these neurophysiological findings would suggest that spatial attention is capable of modulating the *early* stages of visual processing for faces, within ∼300 ms from stimulus onset. Importantly, button-press data are unable to index this early stage of visual processing, as both the response time (RT) and accuracy measures obtained on a given trial necessarily represent the endpoint, or culmination, of target processing. Given that the typical latency for button-press responses (∼500–600 ms) far exceeds the period in which ERP studies have reported modulatory effects of spatial attention in face-classification tasks, it is perhaps not surprising that behavioural studies have thus far failed to observe attentional effects.

**Figure 1:**
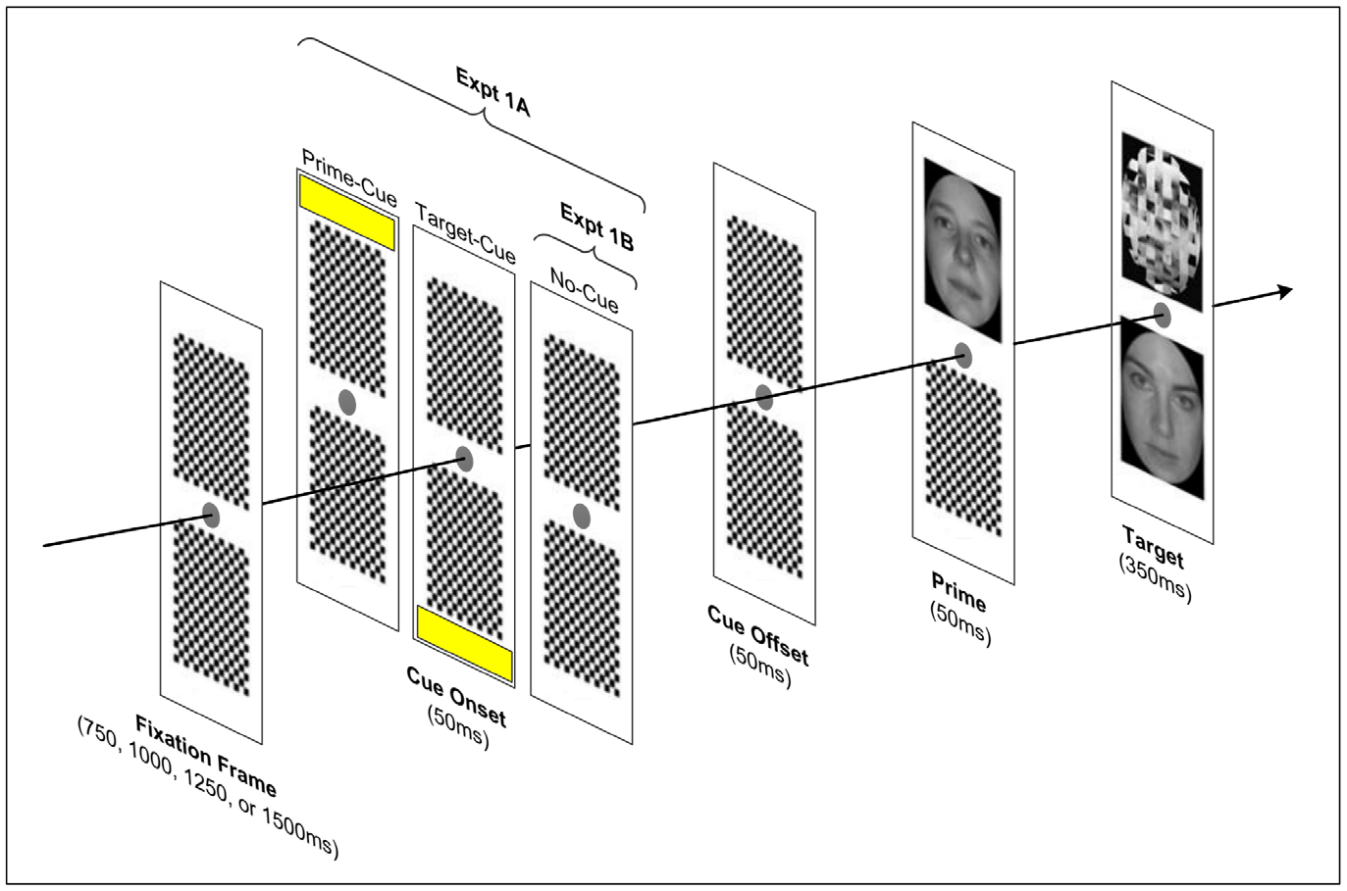
Trial structure for Expts 1A & 1B. Here we depict a congruent trial, in which the prime and target were of the same sex (i.e. female). Each frame consisted of two vertically displaced panels and a central fixation point, presented for identical durations within each frame. Prime and target items always appeared in the upper and lower panels respectively. Both experiments used a variable fixation duration (750 ms–1500 ms); in Expt 1A, the sudden onset and offset of a yellow bar captured participants’ transient spatial attention at the prime (upper) or target (lower) location. The individuals shown here provided written informed consent to the reproduction of their photographs in publication.

In the present study, we sought to document the behavioural complement of these early neurophysiological effects of spatial attention. Our response paradigm was specifically designed to examine face processing during the first 350 ms of stimulus processing. Rather than pressing a button to indicate their response, in our task subjects classified the sex of a target face by reaching out to touch the left or right side of the computer monitor. Importantly, we used a motion-capture device (Optotrak) to sample the position of the hand during the reaching response, which resulted in a high resolution continuous dataset on each trial. There is now a burgeoning literature on the use of such continuous movement measures in cognitive psychology [32]–[36], which are purportedly able to capture dynamic interactions between multiple cognitive processes reflected in motor output [37]. The principal advantage of reaching responses in the present study is that they enable subjects to *begin* their classification response very early without penalty. We required participants to initiate their reaching movement within 350 ms of the target’s onset, ensuring that the initial stages of their classification movements were (frequently) made while subjects were still accumulating evidence about the target. In light of the effects documented in the ERP literature, we reasoned that the attentional effects on face processing would be most visible during this early stage of stimulus processing. We combined this continuous behavioural measure with an adaptation of the masked priming paradigm [38], in which the target face always appeared in the lower of two vertically displaced panels (see Figure 1), and was temporally preceded by a prime face that always appeared in the upper panel. The prime stimuli were either the same sex as the target (i.e. congruent), or of the opposite sex (incongruent), were presented very briefly (50 ms) and immediately backward masked, such that participants were generally unable to report seeing the prime. We assessed the extent to which the masked face was processed by examining how prime-target congruence modulated subjects’ overt response to the target. In button-press versions of this paradigm, participants typically respond faster and more accurately to congruent prime-target pairings than to incongruent pairs, a result termed the Masked Congruence Effect (MCE) [16], [39]–[41]. The MCE thus provides an index of prime processing – the key question here is whether the allocation of attention to the prime’s location (in space or time) modulates prime processing at all (i.e. larger congruence effects for attended primes).

To answer this question, we examined the MCE evident in subjects’ reaching trajectories in the context of manipulations of both spatial attention (Expt 1A) and temporal attention (Expt 1B). In Expt 1A, we adapted a spatial cueing procedure introduced by Lachter and colleagues [25] in which an exogenous cue localised subjects’ transient spatial attention at either the prime or target location (upper or lower panel – see Figure 1). Variants of this paradigm, widespread in the masked priming literature [16], [23], [24], [42], [43], typically yield robust spatial cueing effects. Importantly, Lachter et al. have highlighted that this paradigm actively prevents ‘slips’ of spatial attention, and, by extension, the possibility that experimental effects observed outside the locus of spatial attention “might actually be due to slippage of attention to the supposedly unattended [stimuli]” [43]. We have adhered to the steps recommended by Lachter to prevent attentional slippage by (1) presenting targets in a fixed location, thereby encouraging subjects to direct endogenous attention to the target location; (2) using a sudden onset spatial cue to capture spatial attention exogenously; and (3) presenting prime items briefly (50 ms) to prevent shifts of attention to them before being backward masked [25]. In Expt 1B we examined how the allocation of temporal attention to the prime-target pair modulated face processing. To this end, we took advantage of the fact that subjects can anticipate temporally predictable events with high precision [44] by manipulating the predictability of target onset. The target could occur after one of four fixation durations (900 ms, 1150 ms, 1400 ms or 1650 ms), thereby yielding an increasing conditional probability that the target would appear at a particular moment given that it had not already been presented (i.e. the hazard function). Ghose and Manusell [45] demonstrated that attentional modulation of neural firing rates in the visual cortex (V4) of rhesus monkeys increases as probability of target onset does. In humans, temporal orienting has also been shown to improve perceptual sensitivity (indexed by *d’*) [46]. For example, Westheimer and Ley [47] had subjects make orientation and stereoscopic depth discriminations for stimuli that could occur at either a fixed or random temporal interval. Discrimination thresholds for both stimulus types were significantly lower when subjects could reliably anticipate stimulus onset (fixed interval), suggesting that temporal orienting can enhance perceptual preparation [40], [47]–[50], but see [51].

In keeping with previous findings [15]–[17], we expected to find a masked congruence effect (MCE) in subjects’ reaching trajectories in both experiments irrespective of attentional allocation. Critically however, we hypothesized that our manipulations of spatial (Expt 1A) and temporal (Expt 1B) attention would modulate the MCE and that this modulatory effect would be most apparent in those reaching responses that were initiated within ∼200 ms of stimulus onset. More specifically, we predicted that the MCE would be strongest when subjects’ spatial attention was captured to the prime’s location (Expt 1A), and as participants’ temporal attention became increasingly focused with the increase in the hazard function (Expt 1B).

## General Methods

### Ethics

Experimental protocol was approved by the Human Research Ethics Committee of Macquarie University. All procedures were in compliance with the NH&MRC Australian Code for the Responsible Conduct of Research and the National Statement on Ethical Conduct in Human Research (2007). All participants provided written informed consent prior to partaking in the experiments.

### Participants

Sixteen undergraduate Macquarie University students aged between 19 and 33 years were recruited to participate in Expt 1A. A subsequent group of 16 different students (ages 19–26 years) participated in Expt 1B. All participants were right-handed and financially compensated for their participation.

### Stimuli

Stimuli were greyscale photographs of male and female faces drawn from the Psychological Image Collection at Stirling database (PICS, http://pics.psych.stir.ac.uk/). Six exemplars of each sex were cropped to exclude face contours and adjusted so that their low-level properties were comparable. Five male and five female faces were assigned as targets, with a single exemplar of each sex allocated as the novel prime for that category. These primes only ever appeared under masked conditions, and were never consciously presented as target stimuli. Each finished stimulus subtended 4.2°×3.37° of visual angle from a viewing distance of 68 cm.

### Design

In Expt 1A we used a 2×2×2 fully-crossed factorial design with the factors Cue Presence (present vs. absent), Cue Location (prime location vs. target location) and Prime Type (congruent vs. incongruent). The factor Cue Location was included as a dummy factor on cue-absent trials to ensure an equal number of cue-present and cue-absent trials. To increase uncertainty, we randomly varied the Fixation-Target SOA between 900 ms, 1150 ms, 1400 ms or 1650 ms. The latter manipulation was included as a factor in Expt 1B, as part of a 4×2 fully crossed factorial design (SOA × Prime Type). There were 80 trials per block; in each experiment participants completed one practice block (not analysed), five experimental blocks and a subsequent prime classification block in which we assessed their prime awareness.

### Apparatus & Procedure

All details pertain to both Expts 1A and 1B, see Figure 1 for trial structure differences between the tasks. Participants sat at a rigid table before a 70×39 cm touchscreen monitor fixed 60 cm from the table edge. Throughout testing the monitor displayed peripheral response buttons marked ‘M’ and ‘F’ (side counterbalanced across participants). The stimulus display consisted of two panels (75×100 pixels), displaced vertically around a fixation dot (see Figure 1). On each trial, the prime face appeared for 50 ms in the upper panel before being backward masked; the target face subsequently appeared for 350 ms below fixation. This brief target duration increased the difficulty of the task for subjects, motivating them to direct their attention to the lower panel, *away* from the critical prime stimulus. The trial sequence commenced when the participant depressed a start button aligned with the body midline, 3.5 cm from the table edge. Participants were instructed to lift off the button as soon as the target face appeared in the lower panel, and immediately classify its sex by reaching out to touch the appropriate response button on the left or right edge of the screen. On each trial, we recorded participants’ response initiation time (LiftOff Latency), defined as the time in milliseconds from target onset until the participant released the start button and began their reaching movement. LiftOff Latency serves as a proxy for Target-Viewing Time, in that it reflects the amount of time the participant had to accumulate target evidence prior to commencing their classification response. Whilst this duration varies on each trial, it is critical to note that Target-Viewing Time values are always preceded by 50 ms of exposure to the masked prime stimulus. Importantly, we encouraged participants to initiate their reaching response quickly by giving negative feedback (a loud buzz) and aborting the trial if their LiftOff Latency exceeded 350 ms from target onset. In contrast, reaching responses to classify the target were not speeded, unfolding over ample time (∼3 seconds) for the finger to change direction or correct its course. The stimulus display was controlled using Presentation software (Neurobehavioral Systems); custom software was written to interface the stimulus display with a motion capture device (OptotrakCertus, NDI). This device recorded participants’ reaching trajectories by sampling the position of a small light-emitting diode fixed to the tip of their right index finger at a rate of 200 Hz. This enabled us to record the finger’s position in *xyz* space every 5 ms.

Although we were not directly concerned with ensuring the subliminality of the masked primes, we briefly assessed participants’ awareness of them at the conclusion of the experiment proper. Subjects were informed of the prime’s presence and instructed to complete a final block of trials in which each target classification was followed by an untimed forced-choice identification of the prime on that trial. Subjects indicated which face appeared as the prime by touching one of two faces presented side-by-side (the real prime and a foil).

## Experiment 1A: Spatial Attention

In Expt 1A we used a non-predictive exogenous cue to orient *spatial attention* either toward or away from the prime location. We predicted that masked congruence effects evident in subjects’ classification movements would be stronger for trials on which spatial attention was captured to the prime’s location (prime-cue condition), rather than to the target’s location (target-cue), or when attention remained diffuse over the whole display (no-cue).

### Data Analysis & Results

#### Accuracy

The mean accuracy in target classification averaged across subjects was 99.79%. Accuracy rates were entered into a repeated measures analysis of variance (ANOVA) with the factors Cue Location (prime-cue, target-cue, no-cue) and Prime Type (congruent, incongruent). Results confirmed neither factor influenced participants’ accuracy rates (all p values >.1).

#### LiftOff latency

**Figure 2:**
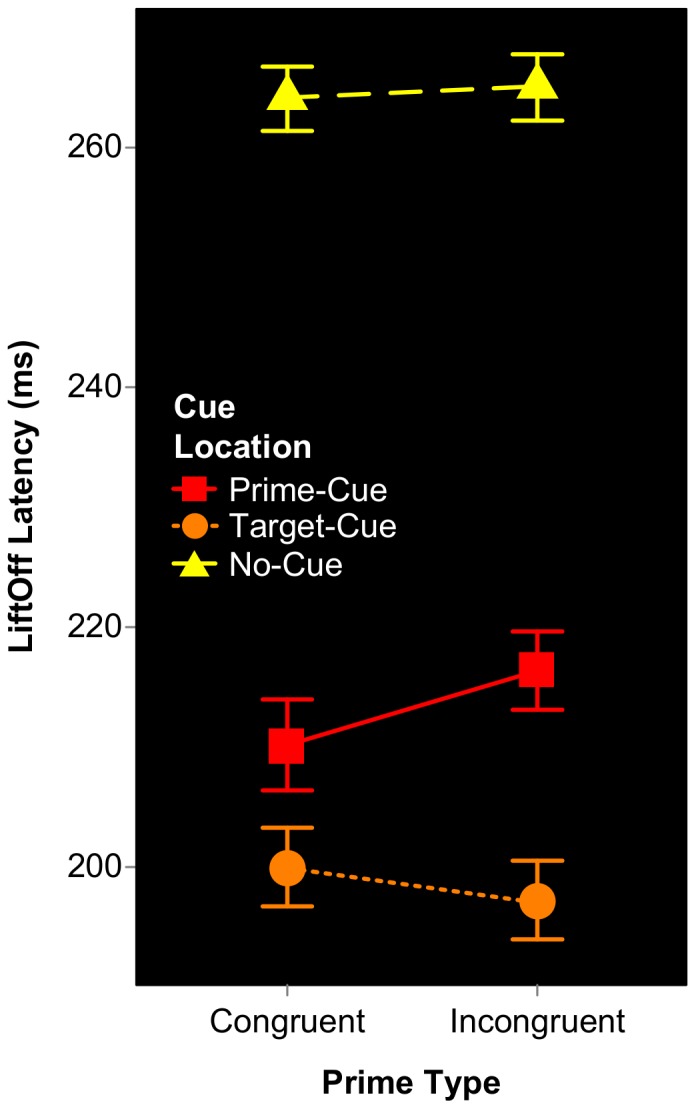
Conditional mean LiftOff Latencies for Expt 1A. Subjects began their reaching movement earliest when the exogenous cue captured attention to the target’s location. Prime Type had no effect on when subjects began their reaching response.

We entered the mean LiftOff Latencies into the same repeated measures ANOVA described above. Here we observed a clear effect of Cue Location, F(2,30) = 130.14, p<.001, in that LiftOff Latencies (see Figure 2) were fastest in the target-cued condition (M = 196 ms), next fastest in the prime-cued condition (M = 210 ms) and slowest in the no-cue conditions (M = 264 ms). All differences were reliable (Tukey HSD; all adjusted p values<0.01), suggesting the spatial cue was effective in localising participants’ spatial attention. In contrast, Prime Type had no effect on LiftOff Latencies (p = .404), nor was the interaction significant (p = .373).

#### Reaching trajectories

We time-normalised reaching trajectories prior to analysis by re-sampling each to produce 100 evenly spaced increments between the point corresponding to 5% of peak tangential velocity and the point at which the finger touched the response button. At each sample we then calculated x-velocity, a signed value indicating the velocity of the finger along the left-right dimension (x-axis). This is the dimension along which subjects indicate their classification response (e.g. “left for male” and “right for female”). Furthermore, because x-velocity is a signed value (positive for movements in the correct direction and negative for movements in the incorrect direction), we assume that this measure can be used as a momentary index of the subject’s response certainty. That is, the more positive x-velocity is, the more quickly the finger is moving in the correct direction. With this measure, congruence effects are typically reflected in higher x-velocities on congruent trials at earlier points in time.

As a consequence of the imposed response initiation deadline in this paradigm, participants must begin their reaching movement before they are really certain which way to go. It is unsurprising then that the quality of this initial classification movement depends on how long subjects viewed the target prior to initiating their response (i.e. LiftOff Latency). There is a positive relationship between these measures, in that the longer participants wait to begin responding, the longer they have to view the target and accumulate evidence about where to reach when they lift off the start button. To exploit this relationship between Target-Viewing Time and x-velocity, we employed a modified version of the Orthogonal Polynomial Trend Analysis (OPTA) procedure developed by Woestenburg [52] and recently adapted by Karayanidis and colleagues [53]. In the present case, the individual trial LiftOff Latencies are used as a covariate in a polynomial regression model of our dependent variable, x-velocity, allowing for a detailed analysis of how reaching responses vary as a function of Target-Viewing Time. The technique has the advantage of being able to estimate x-velocity at the individual trial level, rather than averaging across many trials, consequently improving the signal-to-noise ratio (SNR). For example, Karayanidis et al. [53] reported OPTA improved the SNR by 2.5 times compared to simple averaging.

**Figure 3:**
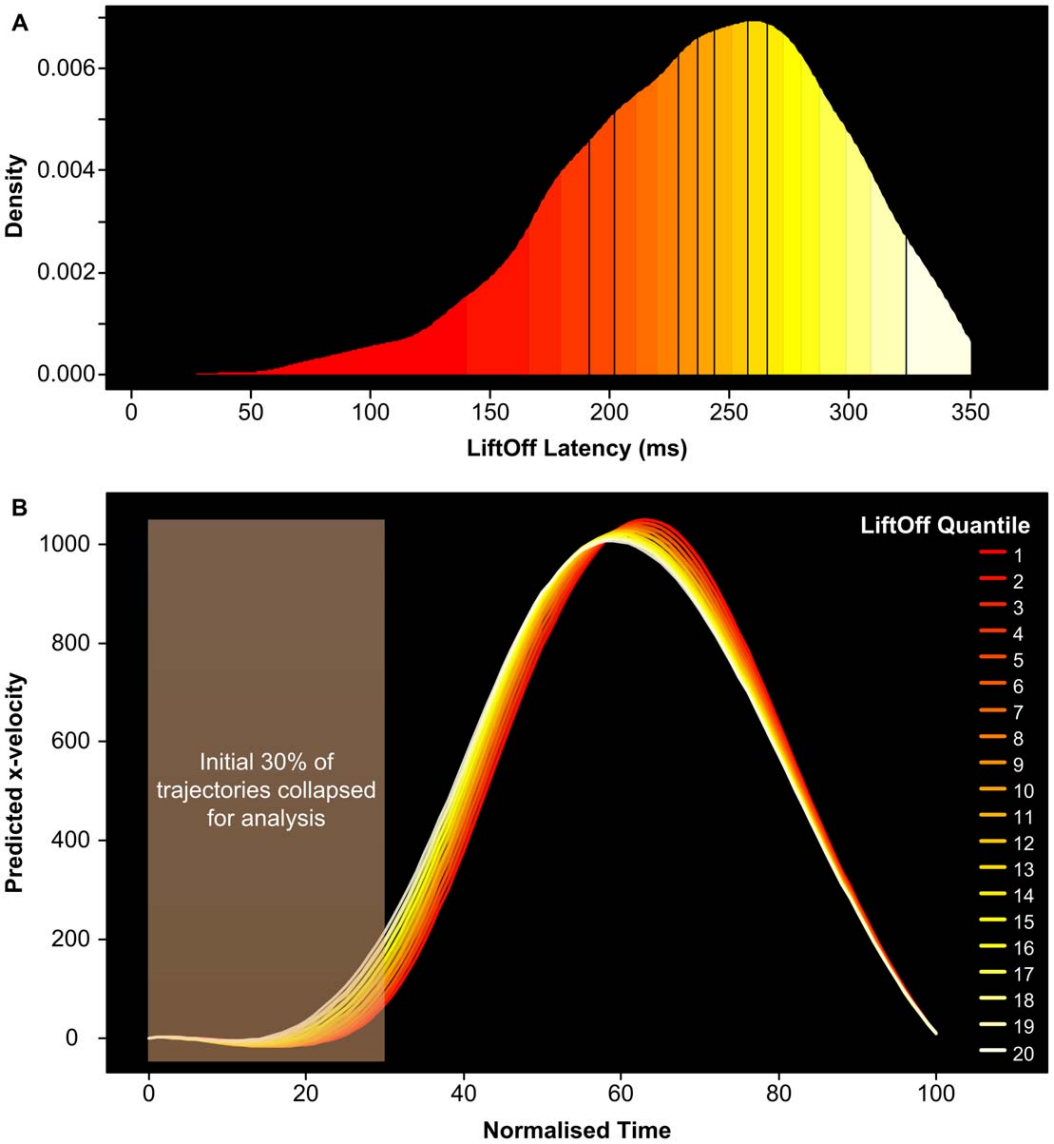
Grouping trajectories by Target-Viewing Time. (A) Analysis begins with the distribution of LiftOff Latencies (i.e. Target-Viewing times), estimated relative to target onset. A modified version of *OPTA* is used to fit a polynomial regression model to the x-velocity profile for each trial. The model includes LiftOff Latency percentile as a covariate (see text). (B) Predicted x-velocity profiles are grouped into semi-decile intervals. Red colours indicate trials with short LiftOff Latencies (beginning at the 1^st^ semi-decile); white-colours correspond to longest LiftOff Latencies (20^th^ semi-decile). Note the clear effect of LiftOff Latency: the longer subjects wait to begin moving, the faster the finger moves in the correct direction.

In the present experiments, the OPTA procedure described below was implemented using custom-software written in R (www.r-project.org). Trials with correct responses in each experimental design cell (i.e. Subject, level of Cue Location, level of Prime Type) were ranked according to their LiftOff Latency, from the shortest (ranked 1st) to longest (ranked *n* ^th^, where *n* is the number of trials for that subject in this design cell). A polynomial regression model was then fitted to the *x*-velocities using LiftOff Latency Rank as the covariate and polynomial terms up to the 6th order. Polynomial terms that did not account for a significant proportion of variance were removed, and the remaining coefficients used to generate *predicted* x-velocity values (one per trial for all subjects). To visualise the effect of Target-Viewing Time on reaching responses predicted trajectories were averaged into semi-decile intervals, resulting in 20 predicted trajectories per experimental condition, per subject (see Figure 3),. The first of these Quantiles represents those trials corresponding to the fastest 5% of LiftOff Latencies; the second represents the next fastest 5% of LiftOffs, and so on. Because we were interested in the participants’ classification responses at the time of movement initiation, we restricted our analysis to the initial 30 samples of the predicted trajectories (i.e. first 30% of the trajectory). We computed the mean x-velocity across this initial portion of the trajectory, resulting in a single value for each trial, which was then submitted to a linear mixed-effects model (LMM) with LiftOff Latency semi-decile included as a fixed effect. Note that although the duration of the initial 30% of trajectories is not uniform across trials (see Figure S2 & S4 in the supplementary materials), we have found that x-velocity is better predicted by the information available at the point of movement initiation (i.e. LiftOff Latency) than total duration (i.e. LiftOff Latency plus the duration of the initial movement). Further details regarding this appear in the supplementary materials.

The OPTA procedure described above yielded 188,480 observations from 16 participants, corresponding to average values for the initial 30% of the trajectory. These data were subjected to analysis using the linear mixed-effect modelling technique (LMM) [54], [55] implemented in R with the lmer4 package [56]. This approach allowed us to simultaneously consider both fixed and random effects in detail (rather than averaging across subjects) and evaluate the contribution of each term to the model by comparing that model with a one that excluded the effect under inspection. In each case, Likelihood tests (AIC & BIC) were used to indicate which model should be preferred. These values provide a measure of two or more models’ relative goodness-of-fit, penalising them for the number of free parameters to prevent over-fitting. We further report coefficients, standard errors (SE), and t-values for the resulting models selected.

Our incremental model comparison procedure resulted in a model that included random slopes between LiftOff Quantile and Subject, together with fixed effects of LiftOff Quantile (1 to 20), Cue Location (prime-cue, target-cue, no-cue), and Prime Type (congruent, incongruent). Each two-way interaction between the latter three fixed effects was similarly verified as significantly improving the model, as well as a final three-way interaction between LiftOff Quantile, Cue Location, and Prime Type. Table 1 presents the regression coefficients, standard errors (*SE*s), and *t* values given by this final model. Here the intercept represents a modified group mean corresponding to the first level of each factor. Thus, the Prime-Cue:Congruent condition forms the reference category from which the other effects deviate. As is typical in LMM analyses [57], [58]–[60], we have taken a coefficient magnitude of at least twice its standard error (i.e. |*t|* >2) as our criterion for significance. For a dataset of the present size, this 2-SE criterion approximates the traditional two-tailed.05 significance level [55].

**Table 1:** Fixed effects for Expt 1A estimated with LMM^a^.

	*b*	*SE*	*t*-val
(Intercept)^b^	−10.928	6.299	−1.74
LiftOff Quantile	2.149	0.390	5.51
Prime Type (incongruent)	−8.585	1.288	−6.67
Cue Location (target-cue)	2.720	1.286	2.11
Cue Location (no-cue)	18.374	1.108	16.58
LiftOff Quantile×Prime Type (incongruent)	−0.684	0.108	−6.35
LiftOff Quantile×Cue Location (target-cue)	−0.465	0.108	−4.32
LiftOff Quantile×Cue Location (no-cue)	−0.129	0.093	−1.39
Prime Type (incongruent)×Cue Location (target-cue)	−5.775	1.825	−3.17
Prime Type (incongruent)×Cue Location (no-cue)	−1.086	1.570	−0.69
LiftOff Quantile×Prime Type (incongruent)×Cue Location (target-cue)	1.344	0.153	8.80
LiftOff Quantile×Prime Type (incongruent)×Cue Location (no-cue)	0.839	0.131	6.39

a LMM: Predicted x-velocity ∼1+ LiftOff Quantile*Prime Type*Cue Location+(1+ Quantile | Subject).

b A modified group mean for the Prime Type (congruent): Cue Location (prime-cue) condition.

**Figure 4:**
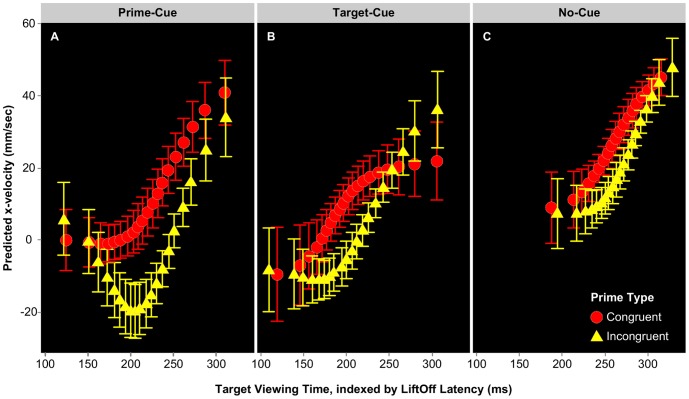
Predicted x-velocity as a function of Target-Viewing Time for each cue condition (A, B, C). Values reflect x-velocity averaged over the initial 30% of the reaching response. Target-Viewing Time (*x*-axis) is the duration for which the subject viewed the target prior to initiating their movement (note this value is always preceded by 50 ms of prime-processing). The slopes clearly indicate that the longer subjects wait to begin their response, the faster they will be moving in the correct direction during the early stages of their movement. Error bars calculated using within-subjects *SE.*

Figure 4 shows conditional mean x-velocity values averaged across the initial 30% of trajectories. X-velocity is shown here as a function of LiftOff Latency, our proxy for Target-Viewing Time. The main effect of LiftOff Quantile is clear, in that the longer subjects take to initiate their response, the faster their finger moves in the correct direction. The expected effect of Prime Type is also present, with incongruent primes producing smaller x-velocity values than congruent primes. With regards to Cue Location, prime-cue trials produced smaller x-velocity values than both target-cue and no-cue trials. An inspection of Figure 4 indicates that these higher x-velocity values associated with the No-Cue condition result because subjects initiate their responses much later on these trials than they do when there is a cue present (see Figure 2). Since they begin responding later in time, subjects have accrued more information about the target by the time they commence their classification response, resulting in more certain movement towards the correct response button.

To ascertain the nature of the significant three-way interaction confirmed in our model comparison procedure, we fitted individual models for each level of Cue Location. These included fixed effects of LiftOff Quantile and Prime Type together with random slopes for LiftOff Quantile×Subject. Here the reference category (intercept) for each model corresponds to the group mean for congruent Prime Type condition. Model comparison and likelihood testing identified the fully interactive model to be preferable for both the prime-cue and target-cue conditions. In both cases we observed significant main effects of LiftOff Quantile (prime-cue: *b = *2.160 mm/sec, *SE* = 0.507 mm/sec, *t* = 4.26; target-cue: *b = *1.655 mm/sec, *SE* = 0.729 mm/sec, *t* = 2.27) and Prime Type (prime-cue: *b* = −8.898 mm/sec, *SE* = 1.217 mm/sec, *t* = −7.31; target-cue: *b* = −14.505 mm/sec, *SE* = 1.179 mm/sec, *t* = 12.30), together with a reliable interaction between these factors (prime-cue: *b* = −0.668 mm/sec, *SE* = 0.102 mm/sec, *t* = −6.56; target-cue: *b* = −0.662 mm/sec, *SE* = 0.099 mm/sec, *t* = 6.71). As can be seen in Figures 4A and 4B, Target-Viewing Time (i.e. LiftOff Quantile) affects priming in both cue conditions, however effects are strongest for the prime-cue condition. In contrast, model comparison for the no-cue condition indicated the additive model to be preferable, resulting in significant main effects of LiftOff Quantile (*b* = 2.073 mm/sec, *SE* = 0.423 mm/sec, *t* = 4.90) and Prime Type (*b* = −7.942 mm/sec, *SE* = 0.452 mm/sec, *t* = −17.58), but no interaction between the two. This suggests priming does not vary as a function of Target-Viewing Time for the no-cue condition (see Figure 4C).

#### Prime detection analyses

Analysis of d′ scores in Expt 1A confirmed the masking procedure was effective. Participants’ mean d’ scores (M = −0.017) did not differ significantly from zero, t(15) = −0.31, p = .76, nor did they differ across levels of Cue Location, F(2, 30) = 1.20, p = .315. This suggests participants’ awareness of the prime was minimal even when their attention was captured at the prime location. Though we agree with those researchers who have suggested that the procedure of regressing subjects’ mean priming effects over their d’ scores often leads to spurious claims of subliminal priming [61], [62], we acknowledge that this is a common practice in the masked priming literature and so we have included this analysis here. To this end, we calculated a Standardised Priming Index (SPI: (incongruent – congruent)/congruent) using participants’ maximum pathoffset values (i.e. peak xy deviation). SPI values were regressed over d’ scores for each level of Cue Location. In all cases, d’ did not significantly predict SPI values (prime-cue: R^2^ = 0.09, F(1, 14) = 1.32, p = .270; target-cue: R^2^ = 0.22, F(1, 14) = 3.90, p = .068; no-cue: R^2^ = 0.005, F(1, 14) = 0.07, p = 0.797). See Figure S1 in the supplementary materials for further details.

### Interim Discussion

The purpose of Expt 1A was to examine the effect of transient spatial attention on the processing of masked faces. The observed results suggest several key points. Firstly, the data highlight the importance of taking stimulus processing time into consideration when examining masked priming effects. For trials on which subjects commence their classification relatively early during face processing (i.e. LiftOff <150 ms from target onset & <200 ms from prime onset), the sex of the masked prime exerts little influence over subjects’ classification of the target face. This is perhaps unsurprising, as at this early stage of evidence accumulation subjects have not yet formulated a strong response to the target for the prime to exert an influence on. Rather, congruence priming effects are strongest when subjects initiate their response between 150–200 ms of Target-Viewing Time. These congruence effects rapidly decrease however, with values for congruent and incongruent conditions converging in the slowest LiftOff Quantiles. This attenuation of congruence effects is clearly evident in the no-cue condition (Figure 4C), in which the priming effect is reduced across all LiftOff Quantiles, due primarily to the comparatively late time at which subjects initiate their movements in this condition.

Secondly, our results are consistent with existing research that suggests the visual system is able to process faces in the near-absence of attention [15], [16], in that our masked face primes influenced subjects’ classification of the subsequent target even when attention was captured at another location. Lastly, although the MCE did not depend on the appropriate allocation of spatial attention, our results provide compelling evidence that spatial attention is indeed capable of modulating the processing of masked faces. By examining the early stages of subjects’ sex-classification movements as a function of Target-Viewing Time, we observed that our manipulation of spatial attention, designed to prevent slips of attention [16], [25], not only facilitated participants’ ability to *initiate* a response to the target, but critically also modulated the extent to which the masked prime affected their target classification movement. Congruence effects were largest when the cue captured spatial attention at the prime’s location.

## Experiment 1B: Temporal Attention

In Expt 1B we investigated the effects of *temporal attention* on masked face processing. The trial structure was identical to that of the no-cue condition in Expt 1A (i.e. no exogenous cues); critically, however, we manipulated the predictability of target onset by varying Fixation-Target SOA. Target onset followed a hazard function [45], occurring after one of four randomly selected durations (900 ms, 1150 ms, 1400 ms or 1650 ms). In this manipulation, participants’ temporal attention to the critical stimuli is optimally focused when target onset is most predictable (at the longer SOAs) and conversely minimally focussed when target onset is least predictable (at the shortest SOA). Accordingly, we expected that the longest SOAs would facilitate masked congruence effects relative to the shorter SOAs. For ease of exposition, we have collapsed these fixation-target intervals into Short SOA (900 ms, 1150 ms) vs. Long SOA (1400 ms, 1650 ms) conditions.

### Results

#### Accuracy

Overall mean sex-classification accuracy for Expt 1B was 99.84%. Mean accuracy rates were entered into a repeated measures ANOVA with the factors SOA (short, long) and Prime Type (congruent, incongruent). Neither of these fixed effects approached significance, F(1,15) = 4.64, p = .995 and F(1,15) = 2.53, p = 1 respectively); their interaction was also not reliable, F(1,15) = 4.71, p = .503).

#### LiftOff latency

**Figure 5:**
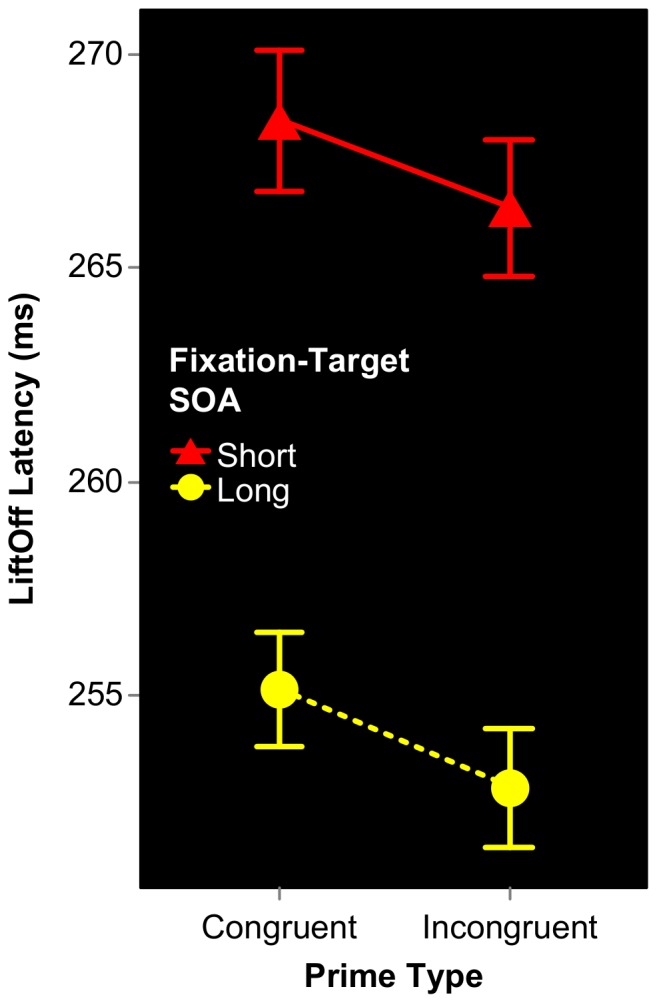
Conditional mean LiftOff Latencies for Expt 1B. Temporal attention modulates LiftOff Latency, in that subjects began their reaching movements earlier when Fixation-Target SOA was long. In contrast, the effect of Prime Type on LiftOff Latency was non-significant.

LiftOff Latencies were entered into the same 2×2 ANOVA described above for Accuracy. As is clear in Figure 5, SOA significantly affected LiftOff Latencies, F(1,15) = 33.34, p<.000, with subjects initiating their responses sooner on Long SOA trials. In contrast, the effect of Prime Type was not significant, F(1,15) = 1.56, p = .231, neither was the SOA×PrimeType interaction, (F<1).

#### Reaching trajectories

Reaching trajectory data for Expt 1B were prepared for analysis using the same OPTA procedures described above. We averaged values across the initial 20% of the OPTA-generated trajectory responses, with the resulting 194,215 observations then subjected to linear mixed-effects modelling and model comparison procedures. It should be noted that the effects reported here are not critically dependent on this selected cutoff of 20%, as directionally similar results were obtained using alternate cutoffs of both 30% and 40%. The preferred model identified using likelihood testing contained random slopes for LiftOff Quantile×Subject, fixed effects of LiftOff Quantile, Prime Type, and SOA, together with their interactions. Critically, the three-way interaction between these factors also significantly improved the model (LiftOff Quantile×PrimeType×SOA. Table 2 presents the regression coefficients, SE and t-values given by this model. As per Expt 1A, effects twice the size of their SE were taken as significant (|t| >2).

**Table 2:** Fixed effects for Expt 1B estimated with LMM^a^.

	*b*	*SE*	*t*-val
(Intercept)^b^	51.536	10.339	4.99
LiftOff Quantile	4.393	0.998	4.40
Prime Type (Incongruent)	−19.461	1.676	−11.61
SOA (Long)	−8.204	1.675	−4.90
LiftOff Quantile×PrimeType (incongruent)	−0.119	0.140	−0.85
LiftOff Quantile×SOA (Long)	0.944	0.140	6.75
PrimeType (incongruent)×SOA (Long)	−21.254	2.370	−8.97
LiftOff Quantile×PrimeType (incongruent)×SOA (Long)	1.481	0.198	7.48

a Model: Predicted x-velocity ∼ LiftOff Quantile*Prime Type*SOA+(1+ Quantile | Subject).

b A modified group mean for the Prime Type (congruent): SOA (short) condition.

**Figure 6:**
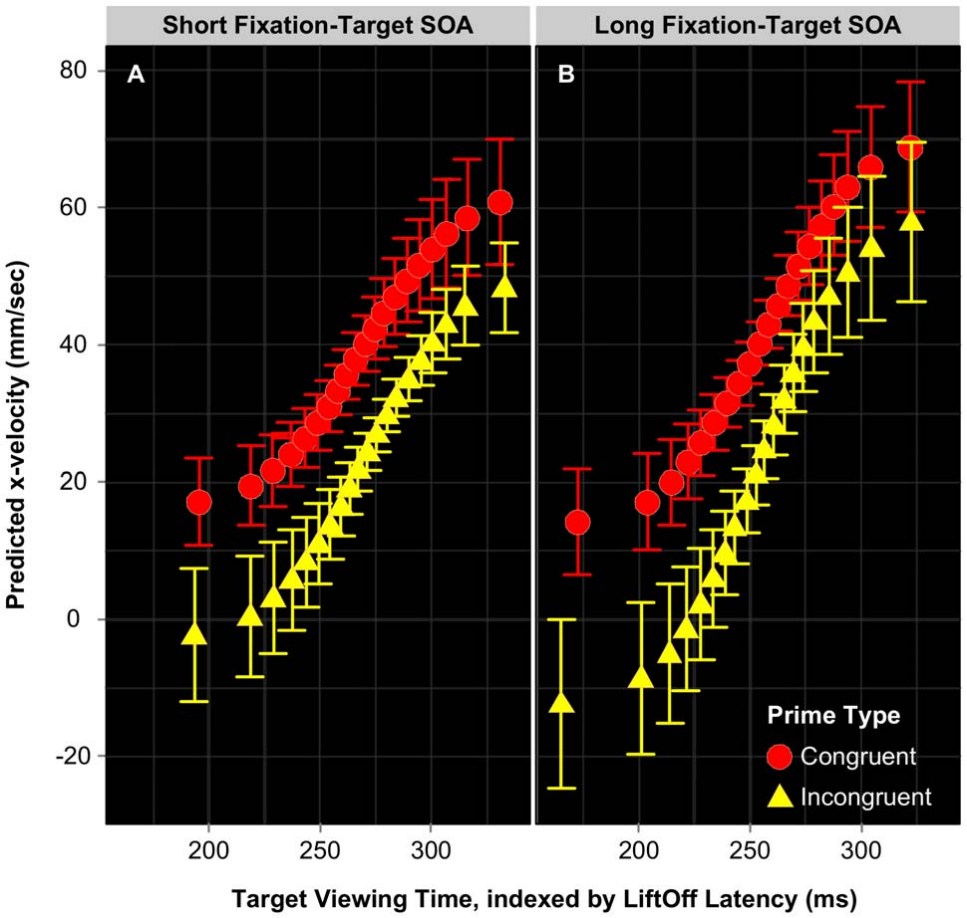
Predicted x-velocity as a function of Target-Viewing Time for short (A) and long SOA (B). All values correspond to an average of the initial 20% of the reaching response. Target-Viewing Time (always preceded by 50 ms of prime processing) positively modulated participants’ classification certainty, such that the longer they waited to begin their reaching response, the faster their finger moved in the correct direction during the early stages of their movement. Error bars calculated using within-subjects *SE.*

To examine the nature of the three-way interaction further, we followed the same procedure as Expt 1A, fitting an individual model for the Short and Long SOA conditions. In each case, we included LiftOff Quantile and Prime Type as fixed effects, together with random slopes for LiftOff Quantile×Subject. In the Short SOA condition, likelihood testing indicated the additive model to be preferable. We observed significant effects of both LiftOff Quantile (*b = *4.325 mm/sec, *SE* = 0.969 mm/sec, *t* = 4.46) and Prime Type (*b* = −20.691 mm/sec, *SE* = 0.806 mm/sec, *t* = −25.68). The absence of the interaction between these factors suggests congruence priming effects are unaffected by Target-Viewing Time (see Figure 6A). In contrast, the preferred model for the Long SOA condition included significant effects of LiftOff Quantile (*b = *5.338 mm/sec, *SE* = 1.161 mm/sec, *t* = 4.59) and Prime Type (*b* = −40.812 mm/sec, *SE* = 1.66 mm/sec, *t* = −24.58), together with a reliable interaction between these factors (*b = *1.372 mm/sec, *SE* = 0.139 mm/sec, *t* = 9.90). In contrast to the Short SOA condition, congruence effects for trials using a longer fixation-target SOA (Figure 6B) are strongest at the earliest Target-Viewing Times, attenuating as Target-Viewing Time increases.

#### Prime detection analyses

Expt 1B d′ scores (M = 0.125) were not significantly different from zero (t(15) = 1.16, p = .26). Additionally, we calculated participants’ mean d′ scores for the short and long SOA conditions and entered these scores into a one-way ANOVA. The effect of SOA was not reliable, F(1,15) = 0.939, p = .348, suggesting that participants’ awareness of the prime stimuli did not increase with their increasing anticipation of an upcoming stimulus. As in Expt 1A, we regressed subjects’ maximum xy pathoffset SPI values (see Expt 1A for details) over d’ scores for each SOA. In both cases, d’ did not significantly predict SPI values (short SOA: R^2^ = 0.02, F(1,14) = 0.32, p = .583; long SOA: R^2^ = 0.11, F(1,14) = 1.74, p = .209). See Figure S2 in the supplementary materials for more details.

### Interim Discussion

In Expt 1B we employed the procedure used by Ghose and Manusell [45] to manipulate temporal attention to the prime-target pair through the use of the hazard function. Target onset could occur after one of four fixation durations, such that the onset of the target was most predictable at the longest fixation-target SOA. Accordingly, we predicted that the MCE would be strongest at this long SOA, as subjects’ temporal attention should be optimally focussed in this condition. There are several key findings suggested by the data. Firstly, we found support for the findings of Expt 1A, in that our masked face primes influenced subjects’ sex-classification responses regardless of how well their attention was focused in time. Secondly, results validated the efficacy of our attentional manipulation, in that subjects initiated their responses fastest when target onset was most predictable. Lastly, and most importantly, we found further evidence to suggest that masked face processing can indeed be modulated by the allocation of attention – in this case, temporal attention. We observed larger and earlier congruence priming effects when subjects were most prepared for the onset of the critical stimuli.

## General Discussion

In the present study we sought to address the current divide between the neurophysiological and behavioural literatures concerning the effects of spatial attention on face processing. Where fMRI studies have documented clear and replicable effects of spatial attention on the neural response for faces [18], [20], [30], [31], [63], the behavioural evidence for this position has been inconsistent [13]–[17], [26]. In the present study, we have reconciled this issue by demonstrating a reliable effect of both spatial and temporal attention on an overt behavioural response to a face target. We employed a behavioural measure designed to index an early stage of stimulus processing thought to be comparable to the time period in which ERP studies have documented modulatory effects of attention on face processing [30], [31], [63] – less than 350 ms from stimulus onset. We here report two key findings from this novel paradigm which, taken together, provide the basis for a more coherent understanding of the relationship between face processing and attention.

Firstly, we have verified that face processing does not *depend* upon the allocation of spatial attention to proceed. This finding replicates and supports previous studies that have reported findings consistent with this claim [15]–[17], [26]. In addition, the results of our second experiment serve to extend this claim to include temporal attention as well, further establishing the unique status of faces within the human visual system. Where masked priming effects elicited by word, letter, and number stimuli are well-documented to rely upon both temporal and spatial attention [23]–[25], [39], [40], [64], here we have demonstrated that masked faces are able to influence the participant’s response to a target stimulus irrespective of both spatial and temporal attention.

Secondly, and more importantly, we have provided compelling behavioural evidence that, while face processing does not depend on focussed spatial or temporal attention, face processing is nevertheless *modulated* by both spatial and temporal attention. Our results in this respect are clear cut – in both the spatial and temporal domain, the allocation of attention to the masked prime stimulus enhanced the masked congruence effect. This critical finding is at odds with much of the existing behavioural research that has failed to find a modulatory effect of spatial attention on face processing [13], [15], [16]. We suspect the key distinction that underlies the discrepancy between these studies and our own is the behavioural response measure employed. Support for this suggestion can be found in a comparison between the present study and one we have reported previously [16]. Using a near-identical trial structure and attentional manipulation, but with an RT measure, Finkbeiner and Palermo found no evidence of attentional modulation of masked face processing. Nevertheless, the very same paradigm in the present study yielded robust effects of attention reflected in subjects’ continuous reaching responses. We suggest that the divergent results obtained with these behavioural measures relate directly to the stage of stimulus processing they are able to index. Responding via a reaching movement allows subjects to *initiate* their categorisation response very early in stimulus processing without penalty, thereby allowing the researcher to observe experimental effects as they emerge in stimulus processing time. In the present case, we were able to document attentional benefits for face processing occurring at less than 200 ms of stimulus processing time. These findings are in accord with ERP studies that document modulatory effects of spatial attention on early visual components elicited by face stimuli (e.g. the N170) [30], [31], [63]. In contrast, button-press responses are cumulative in nature, in that they necessarily reflect the endpoint of stimulus processing. Executed at a latency of around ∼500–600 ms, button-press responses index target processing at a stage that may simply be too late to reveal clear attentional effects on a process as robust as sex-discrimination [13]–[16]. By providing a behavioural measure capable of examining the early stages of stimulus processing, the current study represents a point of coherence between the previously discrepant neuroimaging and behavioural findings concerning the role of spatial attention in face processing.

Although we have shown here that face processing is not impervious to attentional modulation, the fact remains that in contrast to other stimulus types, the visual system prioritises faces such that they may nevertheless be processed outside the focus of attention. What mechanism gives rise to this unique characteristic? We have suggested previously [16] that face-sex discrimination processes, thought to rely on low spatial frequency information that is predominantly carried by magnocellular channels [65], [66], may be supported by a subcortical face processing route that escapes attentional modulation [67], [68]. A recent study using non-face stimuli may provide some support for this claim. Dobromir and colleagues observed implicit processing of peripheral low-level motion coherence when spatial attention was engaged elsewhere [69]. Although the issue remains debated [70], some researchers have suggested that, like face-sex discrimination, the perception of coherent motion may be largely supported by the magnocellular system [71]. Thus, coherent motion may represent one of the only stimulus-types outside of face-sex that the visual system is capable of processing in the near-absence of spatial attention. Research capitalising on this and other stimulus-types favoured by magnocellular channels will be important to pursue to further elucidate the conditions under which attention is able to influence this subcortical route.

### Conclusion

The present study reports two main findings. First, using a novel version of the reach-to-touch paradigm, we have replicated previous findings showing that masked face primes are processed regardless of whether they are spatially attended or not. We have extended this finding by showing that the same positive effect of priming can be obtained when manipulating temporal attention. Second, we have shown for the first time that the masked congruence priming effect obtained with faces is sensitive to manipulations of both spatial and temporal attention. That is, while we found positive masked priming effects for both attended and unattended face primes, our results reveal that these positive priming effects are nevertheless modulated by manipulations of attention. To our knowledge, this is the first study to report an interpretable modulatory effect of attention on the processing of face-sex in a behavioural paradigm. We have suggested that the critical difference between previous studies and ours is the continuous behavioural measure used in the present study. The virtue of the reach-to-touch paradigm is that it allows subjects to initiate their response with impunity very early on in stimulus processing. This, in turn, provides the opportunity to observe the emergence of effects (and their modulations) within the first few hundred milliseconds of stimulus-processing time. This level of temporal resolution is not available in discrete behavioural measures (e.g. button presses), which have been used previously.

## Supporting Information

Figure S1 **Correlation of the MCE and** ***d’*** **for prime-cue (A), target-cue (B), and no-cue (C) trials.** To assess whether the MCE in Expt 1A systematically varied with prime visibility, we calculated a Standardised Priming Index (SPI) using peak *xy* deviation, and regressed SPI over participants’ *d’* scores for each cue condition. The relationship did not reach significance for any of the cue conditions.
(TIF)

Figure S2 **Conditional mean durations of the initial 30% of Expt 1A trajectories, shown as a function of Target-Viewing Time.** Our primary analysis for Expt 1A examines how *x*-velocity (averaged over the initial 30% of trajectory responses) varies with Target-Viewing Time (i.e. LiftOff Latency). Here we present the conditional mean *duration* of this initial 30%. Duration is clearly affected by Target-Viewing Time, in that earlier LiftOff Latencies result in longer trajectory durations and, thus, the initial 30% spans a longer period of time. Since duration varies between trials then, one might think that duration might affect *x*-velocity during the initial 30%, and that it should therefore be incorporated into our primary analysis. To establish whether this is the case, we compared a Linear Mixed Effects Model (LMM) that included LiftOff Latency as a predictor of initial x-velocity (Model 1) with Model 2 that substituted LiftOff Latency for Total Duration (i.e. LiftOff Latency+Duration of Initial 30%). Both models have the same number of parameters. If Total Duration is a better predictor of initial x-velocity, then Model 2 should provide a better fit to the data. However, AIC, BIC, and Log Liklihood comparisons indicated that the predictive power of Model 2 was no better than that of Model 1. This finding suggests that the initial reaching movement is no more strongly influenced by the information that is accumulated during the initial movement as it is by the information present at the beginning of the initial movement. For this reason, we have chosen to depict initial x-velocity as a function of LiftOff Latency as opposed to Total Duration.
(TIF)

Figure S3 **Correlation of the MCE and** ***d’*** **for short SOA (A) and long SOA (B) trials.** As in Expt 1A, we assessed the relationship between the MCE and prime visibility by regressing a Standardised Priming Index (SPI) over *d’* values for each level of SOA. *d’* did not significantly predict SPI in either case.
(TIF)

Figure S4 **Conditional mean durations of the initial 20% of Expt 1B trajectories, shown as a function of Target-Viewing Time.** As in Expt 1A, we inspected the conditional mean durations of the selected analysis period for Expt 1B (initial 20% of trajectories). Again, duration decreases as a function of Target-Viewing Time, with later LiftOff Latencies corresponding to shorter durations for the initial 20% of the response. To assess whether the total duration (LiftOff Latency+duration of initial 20%) was a better predictor of initial x-velocity than just LiftOff Latency, we compared a Linear Mixed Effects Model (LMM) that included LiftOff Latency with a model that substituted this term with Total Duration (both models have the same number of parameters). As in Experiment 1A, AIC, BIC, and Log Liklihood comparisons favoured Model 1, suggesting LiftOff Latency to be a better predictor of initial *x*-velocity than total duration (LiftOff Latency+duration of initial 30%).
(TIF)
