# Supplementary figures and images for: Spatial and Temporal Attention Modulate the Early Stages of Face Processing: Behavioural Evidence from a Reaching Paradigm
Source: PLoS One. 2013 Feb 28;8(2):e57365. doi: 10.1371/journal.pone.0057365 (PMC3585364; doi:10.1371/journal.pone.0057365)

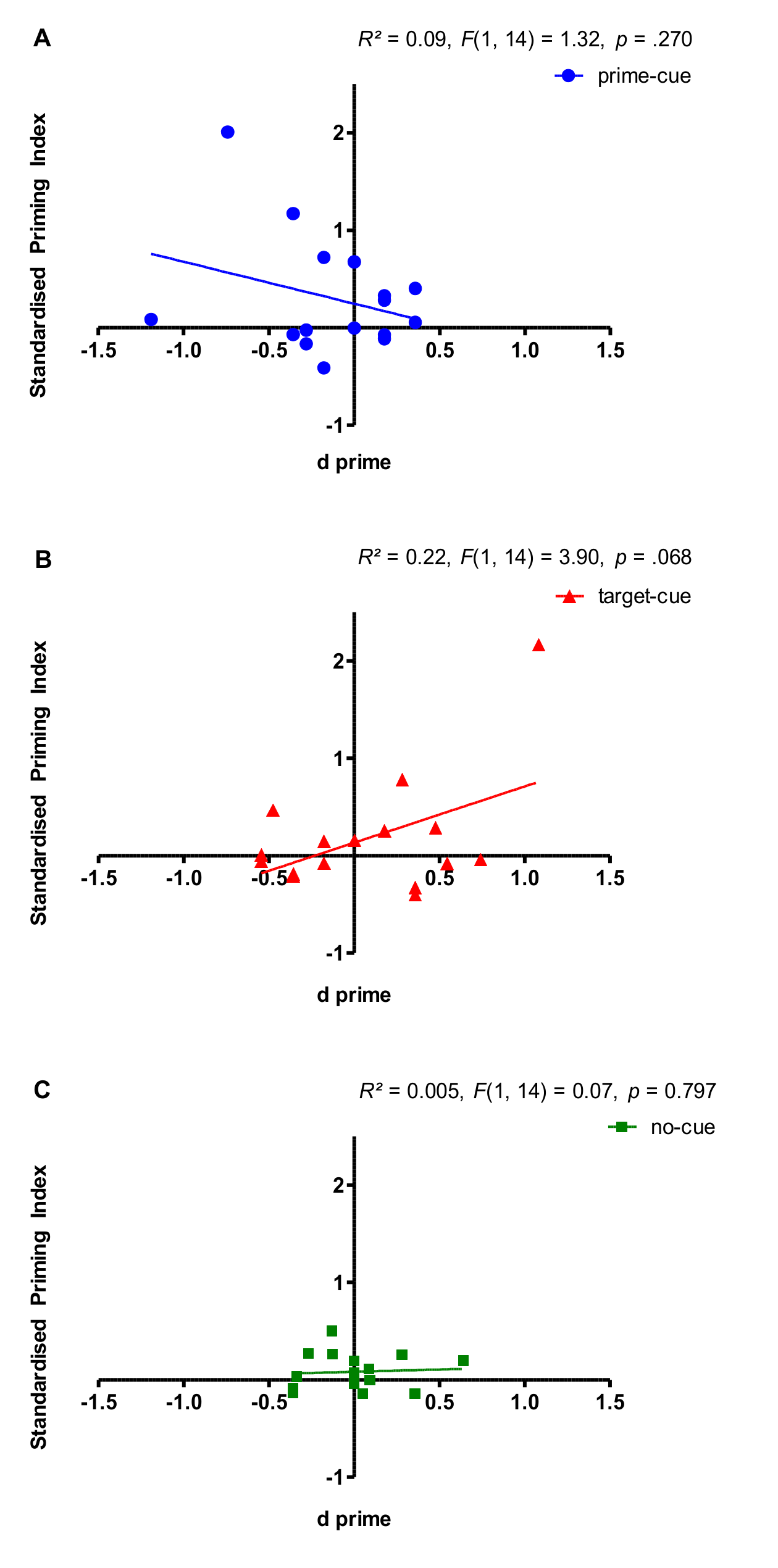

Supplement: Figure S1 — Correlation of the MCE and d’ for prime-cue (A), target-cue (B), and no-cue (C) trials. To assess whether the MCE in Expt 1A systematically varied with prime visibility, we calculated a Standardised Priming Index (SPI) using peak xy deviation, and regressed SPI over participants’ d’ scores for each cue condition. The relationship did not reach significance for any of the cue conditions. (TIF) [file pone.0057365.s001.tif]

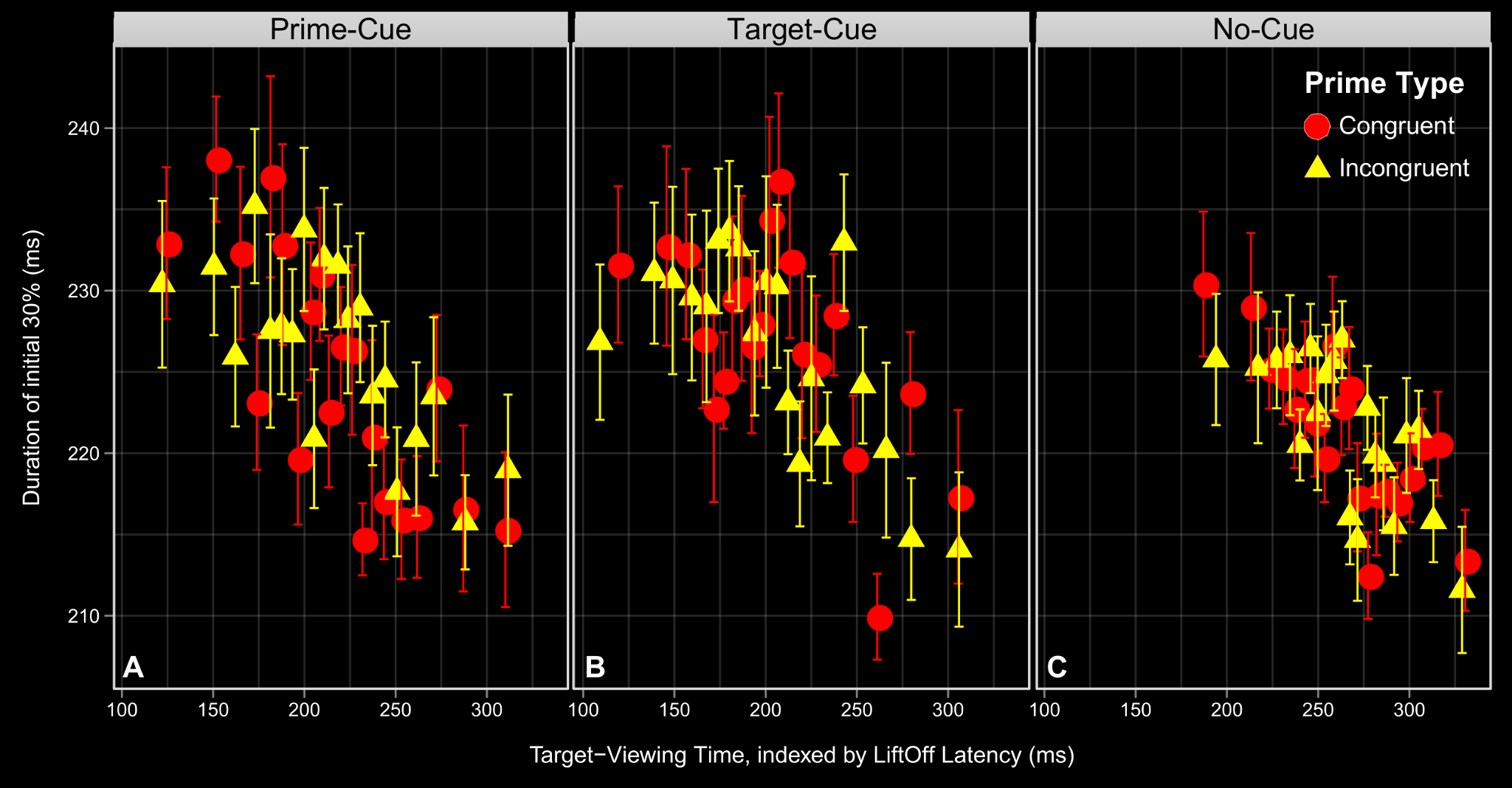

Supplement: Figure S2 — Conditional mean durations of the initial 30% of Expt 1A trajectories, shown as a function of Target-Viewing Time. Our primary analysis for Expt 1A examines how x-velocity (averaged over the initial 30% of trajectory responses) varies with Target-Viewing Time (i.e. LiftOff Latency). Here we present the conditional mean duration of this initial 30%. Duration is clearly affected by Target-Viewing Time, in that earlier LiftOff Latencies result in longer trajectory durations and, thus, the initial 30% spans a longer period of time. Since duration varies between trials then, one might think that duration might affect x-velocity during the initial 30%, and that it should therefore be incorporated into our primary analysis. To establish whether this is the case, we compared a Linear Mixed Effects Model (LMM) that included LiftOff Latency as a predictor of initial x-velocity (Model 1) with Model 2 that substituted LiftOff Latency for Total Duration (i.e. LiftOff Latency+Duration of Initial 30%). Both models have the same number of parameters. If Total Duration is a better predictor of initial x-velocity, then Model 2 should provide a better fit to the data. However, AIC, BIC, and Log Liklihood comparisons indicated that the predictive power of Model 2 was no better than that of Model 1. This finding suggests that the initial reaching movement is no more strongly influenced by the information that is accumulated during the initial movement as it is by the information present at the beginning of the initial movement. For this reason, we have chosen to depict initial x-velocity as a function of LiftOff Latency as opposed to Total Duration. (TIF) [file pone.0057365.s002.tif]

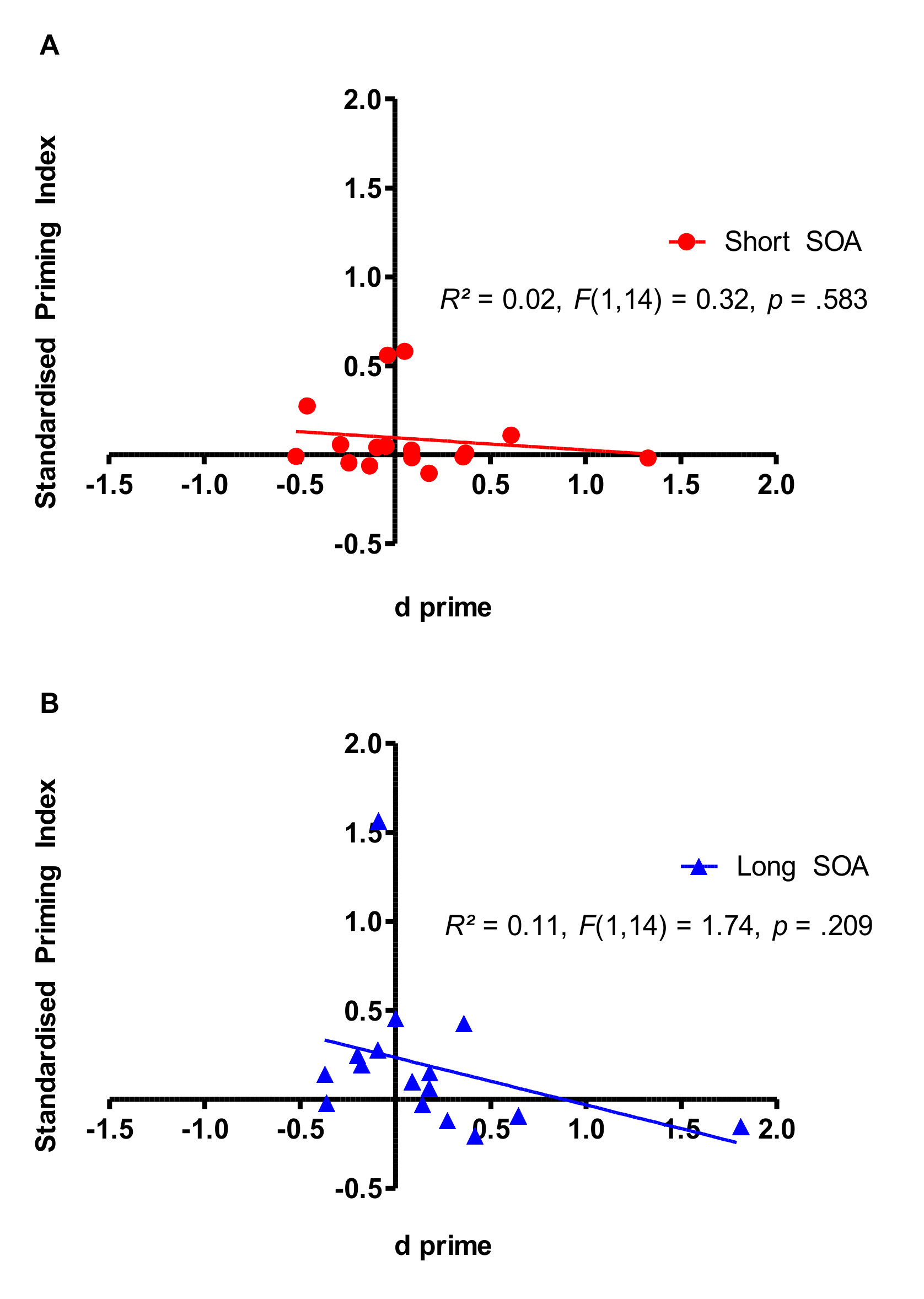

Supplement: Figure S3 — Correlation of the MCE and d’ for short SOA (A) and long SOA (B) trials. As in Expt 1A, we assessed the relationship between the MCE and prime visibility by regressing a Standardised Priming Index (SPI) over d’ values for each level of SOA. d’ did not significantly predict SPI in either case. (TIF) [file pone.0057365.s003.tif]

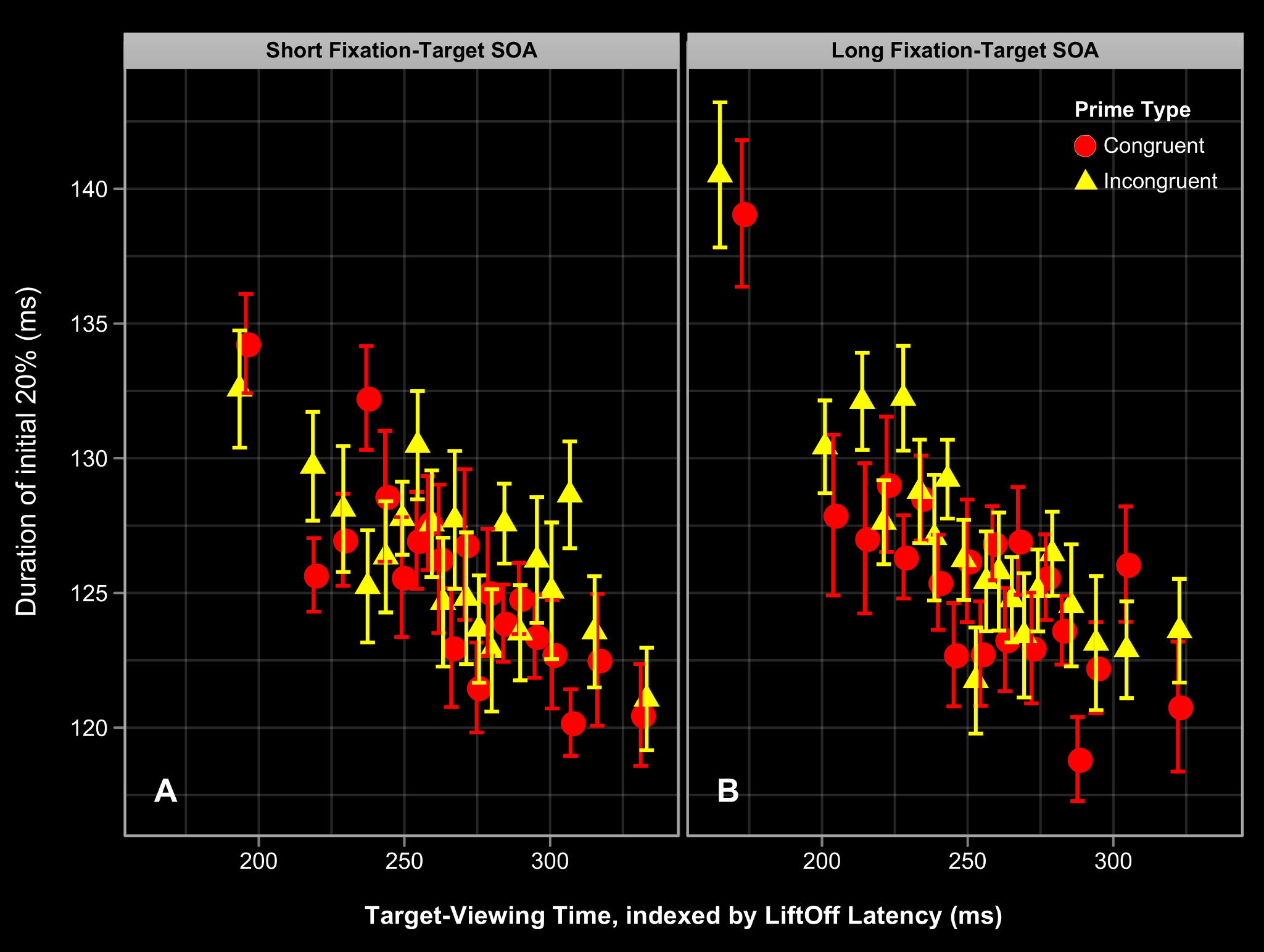

Supplement: Figure S4 — Conditional mean durations of the initial 20% of Expt 1B trajectories, shown as a function of Target-Viewing Time. As in Expt 1A, we inspected the conditional mean durations of the selected analysis period for Expt 1B (initial 20% of trajectories). Again, duration decreases as a function of Target-Viewing Time, with later LiftOff Latencies corresponding to shorter durations for the initial 20% of the response. To assess whether the total duration (LiftOff Latency+duration of initial 20%) was a better predictor of initial x-velocity than just LiftOff Latency, we compared a Linear Mixed Effects Model (LMM) that included LiftOff Latency with a model that substituted this term with Total Duration (both models have the same number of parameters). As in Experiment 1A, AIC, BIC, and Log Liklihood comparisons favoured Model 1, suggesting LiftOff Latency to be a better predictor of initial x-velocity than total duration (LiftOff Latency+duration of initial 30%). (TIF) [file pone.0057365.s004.tif]
